# Development and Evaluation of Real-Time Reverse Transcription Recombinase Polymerase Amplification Assay for Rapid and Sensitive Detection of West Nile Virus in Human Clinical Samples

**DOI:** 10.3389/fcimb.2020.619071

**Published:** 2021-02-23

**Authors:** Priyanka Singh Tomar, Sanjay Kumar, Sapan Patel, Jyoti S. Kumar

**Affiliations:** ^1^ Division of Virology, Defence Research and Development Establishment, Gwalior, India; ^2^ Division of BDTE, Defence Research and Development Establishment, Gwalior, India; ^3^ School of Studies in Botany, Jiwaji University, Gwalior, India

**Keywords:** Env, flaviviruses, real-time reverse transcription recombinase polymerase amplification, rapid detection, West Nile virus

## Abstract

West Nile virus (WNV) causes West Nile fever and encephalitis worldwide. Currently, there are no effective drugs or vaccines available in the market to treat WNV infection in humans. Hence, it is of paramount importance to detect WNV early for the success of the disease control programs and timely clinical management in endemic areas. In the present paper, we report the development of real-time reverse transcription recombinase polymerase amplification (RT-RPA) assay for rapid and real-time detection of WNV targeting the envelope (env) gene of the virus. The RPA reaction was performed successfully at 39°C for 15 min in a real-time thermal cycler. The sensitivity of this assay was found similar to that of the quantitative real-time RT PCR (RT-qPCR) assay, which could detect 10 copies of the gene. The efficacy of the assay was evaluated with a panel of 110 WN suspected human samples showing the signs of retinitis, febrile illness and acute posterior uveitis. In comparison with RT-qPCR, RT-RPA showed a specificity of 100% (CI, 95.07–100%) and sensitivity of 96.15% (CI, 80.36–99.90%) with a negative (NPV) and positive predictive value (PPV) of 98.65 and 100%, respectively. The level of agreement between RT-RPA and reference RT-qPCR assay was shown to be very high. The turnaround time of real-time RPA assay is about 10-20 times faster than the RT-qPCR, which confirms its utility in the rapid and sensitive diagnosis of WNV infection. To the best of our knowledge, this is the first report which deals with the development of real-time RT-RPA assay for simple, rapid, sensitive, and specific detection of WNV in human clinical samples. The present RT-RPA assay proves to be a powerful tool that can be used for the rapid diagnosis of a large number of patient samples in endemic settings.

## Introduction

WNV infection is responsible for causing acute febrile illness in horses, birds, and humans. (Cao et al., 2016). WNV has caused worldwide outbreaks including Africa, America, and India. WNV major cases reported during 1999 in New York and 2014 in Texas (Martinez et al., 2017), which globally caused high morbidity and mortality rate. In the USA, in 2019, a total of 49 states have recorded WNV cases and overall, 2,544 cases of WNV have been reported to the CDC (Centers for Disease Control and Prevention, 2019). Out of these, 1,594 (63%) were classified as neuroinvasive cases (such as meningitis and encephalitis) and 950 (37%) were detected as non-neuroinvasive. The first case was reported in Uganda’s West Nile region in 1937 which has later become an important epidemic in many parts of the world (Chancey et al., 2015). The WNV belongs to Japanese encephalitis (JE) virus serocomplex and is related to the Dengue fever virus (DENV), Murray Valley encephalitis virus (MVEV) and Saint Louis encephalitis virus (SLEV). It is an arthropod-borne virus with the family *Flaviviridae* and genus Flavivirus. The WNV has a single-stranded, positive-sense RNA genome (Mukhopadhyay et al., 2003; Solomon et al., 2003; Parida et al., 2004; Lim et al., 2011; Colpitts et al., 2012; Cao et al., 2016). The genome comprises a single ORF with a size of about 11 kb without a polyadenylation tail at the 3′end (Friebe and Harris, 2010). The RNA of WNV is translated into a single polyprotein which is further cleaved by host and viral proteases as three structural proteins, namely, capsid (C), envelope (E), membrane (M) protein, and seven non-structural proteins viz., NS1, NS2A, NS2B, NS3, NS4A, NS4B, and NS5 (Knipe and Howley, 2001; Aberle et al., 2018).

Phylogenetic analysis of WNV has revealed that it can be classified into nine genetically distinct lineages (Pachler et al., 2014). Lineage 1, is widespread and further classified into lineage 1a, which includes the American strains (Lanciotti et al., 1999), Lineage 1b (Kunjin virus), which mainly exists in Australia, and Lineage 1c was identified from India (Bondre et al., 2007). Lineage 2 isolates have been detected from Africa, including South Africa and parts of Europe such as Greece, Hungary, and Italy (Papa et al., 2010). In India, the human WNV infection was reported for the first time in 1952 from Bombay (Banker, 1952). WNV outbreak has been currently reported in Indian states including Kerala and Tamil Nadu in 2011 (Anukumar et al., 2011; Shukla et al., 2012). The serological evidence of WNV infection has also been reported from Madhya Pradesh, Maharashtra, Andhra Pradesh, Karnataka, Rajasthan, and Orissa (Paramasivan et al., 2003; Friebe and Harris, 2010; Khan et al., 2011).

There are various laboratory-based methods available for the diagnosis of WNV infection. Serologically, an infection can be inferred by the presence of virus-specific neutralizing and immunoglobulin M (IgM) antibodies. The collection of specimens within one week of illness may not be positive for WNV-specific IgM and hence should be repeated. A positive test for WNV-specific IgG without a positive WNV IgM is suggestive of a past infection of Flavivirus. Virus confirmation is based on virus isolation and by plaque reduction neutralization (PRNT) test. But both methods are laborious and time-consuming, requiring at least 6 to 7 days for completion (Martin et al., 2000; Shukla et al., 2012).

Additionally, several PCR-based detection techniques have been available for the detection of WNV which includes conventional and real-time RT-PCR-based assays (Vanlandingham et al., 2004; Kumar et al., 2014) but, these assays require well-trained personnel and expensive equipment which precludes its application in the field settings. There are also a number of isothermal gene amplification techniques have been developed during past decades such as nucleic-acid-sequence-based amplification (NASBA) (Compton, 1991), loop-mediated isothermal amplification (LAMP) (Notomi et al., 2000), helicase-dependent amplification (HDA), cross-priming amplification (CPA) and self-sustained sequence replication reaction (3SR) (Gupta et al., 2017). However, all these isothermal amplification methods have their own limitations including real-time PCR-based assays requiring an expensive instrument for amplification (Compton, 1991; Chan and Fox, 1999). LAMP assay requires 6 sets of primers for amplification. HDA requires an enzyme, DNA helicase for denaturation of DNA double helix and the assay completes in 90 min. The CPA requires complex primer designing and 3SR is dependent on various incubation temperatures. In recent years, rapid isothermal gene amplification technique namely recombinase polymerase amplification (RPA) has been used as a preferred alternate to PCR.

RPA assay is much more rapid than any other existing technique and required only 15-20 min to complete the reaction and are highly specific and sensitive. The RPA technique utilized three key enzymes: a recombinase (*E.coli* Rec A), a strand displacing DNA polymerase enzyme, a single-stranded DNA binding protein (SSB); an accessory protein required for the completion of the RPA reaction process (Piepenburg et al., 2011). RPA assays are performed at an isothermal temperature (37–42°C) and results can be easily analyzed through real-time monitoring. In RPA reaction the recombinase and loading factor synthesize a nucleoprotein filament that binds with probe and single-stranded primers, which scan the whole double-stranded DNA for homologous sequences. After homology is searched, the filament inserts into the dsDNA, synthesizing a D-loop like structure and DNA strands become separated. The SSBs stabilizes the separated DNA strands and the primers hybridize with the target strand. Afterward recombinase disassembled from the nucleoprotein filament and allows elongation of primers by strand-displacing DNA polymerase. Newly synthesized DNA strands are used for exponential amplification of the RPA cycle (Hill et al., 2014). Alternatively, it is also possible to amplify RNA targets using a reverse transcriptase enzyme (Wahed et al., 2015).

In the absence of effective vaccines or drugs, it is of paramount importance to detect WNV early for the success of the disease control programs and timely clinical management in endemic areas. The present study describes the development of a rapid real-time RT-RPA assay for sensitive and accurate detection of WNV in human clinical samples.

## Materials and Methods

### Cells, Virus Strains, and Extraction of Viral RNA

Vero cells were procured from National Centre for Cell Science (NCCS) Pune, India, and were maintained in complete Eagle Dulbecco’s minimum essential medium (Gibco, Invitrogen, USA) and antibiotic-antimycotic solution (50 mg/ml penicillin and streptomycin) (Sigma, USA). Cells were incubated at 37°C with 5% CO_2_ required for normal growth. The WNV (Eg101 strain, GenBank accession number AF260968) was received from the Institute of Tropical Medicine, Nagasaki, Japan. WNV was propagated by continuous subculturing in C6/36 cell lines. The virus titer was determined through plaque assay in Vero cells according to standard protocol (Igarashi et al., 1994). The viruses utilized in the present study for cross-reactivity study include the Yellow fever virus, Dengue virus, Japanese Encephalitis virus, Saint Louis Encephalitis virus, Chikungunya virus, Ross River virus, Measles virus, Mumps virus, and Rubella virus (**Table S1**). The viral RNA was isolated from an aliquot (140 μl) of WNV infected cell culture supernatant and patient’s samples (plasma and serum) employing QIAamp Viral RNA Mini Kit (Qiagen, Germany), as per the manufacturer’s instructions. The eluted RNA was stored at -80°C until further use.

### Designing of Primers and Probe

The RPA probe and primers were designed manually according to the guidelines of RPA as mentioned in the Twist Dx manual (Twist Dx, UK). Four sets of the Basic RT-RPA primers were designed to target the env gene sequence. The probe was designed within the amplification region of the best primer set for the development of the RT-RPA assay. The primers were procured from Eurofins MWG Operon (Bangalore, India), while the probe was synthesized by Biosearch Technologies, Inc. (USA). The probe contains an oligonucleotide backbone that consists of 6-fluorescein amidite (FAM) as fluorophore and Black Hole Quencher (BHQ-1) as a quencher, while a THF site located between FAM and BHQ-1. The probe is blocked from polymerase extension by a 3′ modification group such as C3 spacer. The details of the oligonucleotide primers and probe used in the present study are given in **Tables 1** and **2**, respectively.

**Table 1:** Details of Env gene specific oligonucleotide primers used for basic reverse transcription recombinase polymerase amplification (RT-RPA) assay.

Name of Primers	Genome Position	Primer Sequences (5’-3’)
Set1 F	6–120	ACTGCCTTGGAATGAGCAACAGAGACTTCT
Set1 R	91–120	GTAGGCTTGTCCTTAGACATGATGGTCACA
Set2 F	325–354	TGGTAAAGGAAGCATTGACACATGCGCCAA
Set2 R	438–467	GGTAGTTTCCATGCGACTCCACAGTGGTTG
Set3 F	449–478	GAGTCGCATGGAAACTACCCCACACAGATT
Set3 R	569–578	ATTGGTGTCAATCCCTGATCGTGGTTCAGA
Set4 F	521–550	CCTTCATACACACTAAAACTTGGAGAGTAT
Set4 R	627–656	TAAACCACTCACGATGGACCAAGAACGTCT

**Table 2:** Sequences of the recombinase polymerase amplification (RPA) primers and probe used for real-time reverse transcription (RT)-RPA study.

Name of Primers	Genome Position	Primer Sequences (5’-3’)
WNV Forward Primer (Set4 F)	521–550	CCTTCATACACACTAAAACTTGGAGAGTAT
WNV Reverse Primer (Set4 R)	627–656	TAAACCACTCACGATGGACCAAGAACGTCT
WNV RPA Probe	567–616	5’ACTGTGAACCACGATCAGGGATTGACACCAA[FAM-dT]G[THF]A[BHQ1-dT]ACTACGTGATGACT-C3-spacer

### Clinical Samples

The WN suspected plasma and serum samples (n = 110) from patients with retinitis, uveitis, and febrile illness (between day 1 and day 9) were used in this study. These samples were provided by the Aravind Eye Hospital, Madurai (Tamil Nadu) India, for the evaluation purpose. The present study was approved by the Institutional Ethical Committee (IBSC/12/VIR/JSK/18).

### Basic RT-RPA Assay

For the development of real-time RT-RPA assay, four sets of primers were designed for the identification of the best one. All four primer sets were tested using a TwistAmp Basic RT kit (Twist Dx, UK). The RT-RPA assay (50 μl) was performed using freeze-dried reagents, 29.5 μl rehydration buffer (Twist Dx, Cambridge, UK), 2.4 μl of each primer (400 nM), 11.7 μl nuclease-free water. All reaction components except template and magnesium acetate were added in a master mix, followed by which it was suspended into reaction tube comprising a dried enzyme pellet; template 1.5 μl (40 ng) and 2.5 μl (280 mM) magnesium acetate was added into the lid of the reaction tubes and properly mixed. The reaction was incubated using temperature gradient 39°C, 40°C, and 41°C and performed for 10, 20, and 30 min in a water bath. During incubation, reaction components were mixed after 5 min of amplification by gentle mixing reaction tubes three to four times and re-incubated the tubes for completion of the reaction. The RPA amplicons were cleaned-up using a QIAquick PCR Purification Kit (Qiagen, Germany) as per the manufacturer’s protocols.

The purified products were checked on 2% agarose gel (Himedia, India) and visualized under the UV gel Doc imaging system (Biorad, USA).

### Real-Time RT-RPA Assay

The real-time RT-RPA reactions were carried out in a 50-μl volume employing the TwistAmp RT Exo kit (Twist Dx, UK) which contains all reagents and enzymes (lyophilized pellets) required for the assay. The optimal primers and probe concentration was determined after running the assay with different concentrations ranging from 100-500 nM. The RT-RPA assay components included 29.5 μl rehydration buffer (Twist Dx, Cambridge, UK), 2.1 μl of each primer (400 nM), 0.6 μl probe (200 nM), and 11.7 μl nuclease-free water. All reagents except the two (magnesium acetate and template) were added in a reaction mix, and were dispensed into each 200 μl reaction tube containing dried reagents; 2.5 μl (280 mM) magnesium acetate and template was added into the lid of the reaction tubes post addition of 1.5 μl (40 ng) to trigger the reaction at 39°C for 15 min. The assay was performed using ABI StepOne Real-time PCR system (Applied Biosystems, USA) and the fluorescence signal was detected in the FAM channel (Excitation 470 nm, Emission 520 nm) in 30 cycles for 30 s. As the RPA assay was performed in a real-time PCR machine, the threshold cycle (Ct) was converted to reaction time (Rt, in min) using the application of a time multiplier. Since the fluorescence was measured every 30s in the RPA reaction; therefore, Rt value is expressed as equal to two times Ct values (i.e., Rt=2Ct [min]). A cut-off value of 1,000 (fluorescence signal at the Y-axis) after 15 min of amplification was established to distinguish positive from negative results.

### Analytical Sensitivity of Real-Time RT-RPA Assay

To determine the analytical sensitivity of RT-RPA, *In-vitro* transcription (IVT) was performed. Briefly, 1.5 kb full-length env gene was cloned in pET 28(a) vector. The recombinant plasmid was extracted using the QIAprep Spin Miniprep kit (Qiagen, Germany). The integrity of the recombinant plasmid was confirmed by env gene-specific RT-PCR. *In-vitro* RNA was synthesized by the Megascript-T7 transcription kit (Invitrogen, USA) using RT-PCR amplified product as the template according to the manufacturer’s instructions. IVT-RNA was quantified using a Nanodrop ND-1000 spectrophotometer (Thermo Scientific, Germany).

Number of copies was determined using the following formula:

$$\text{Y molecules}/\mu \text{l}=(\text{X g}/\mu \text{l RNA}/[\text{transcript length in nucleotides }\times 340])\times 6.022\times 10^{23}$$

The sensitivity of real-time RT-RPA assay was evaluated in terms of RNA copy number by 10-fold serial dilutions of the RNA transcript and it was used for the generation of the standard curve. The RNA of Eg101 strain of WNV and nuclease-free water was served as positive and negative control respectively in each run.

### TaqMan Real-Time RT-PCR

The CDC reported quantitative real-time RT-PCR (RT-qPCR) was employed as the reference molecular detection method (Lanciotti et al., 2000) and for the comparison of the sensitivity of the real-time RT-RPA assay. The RT-qPCR was performed on the ABI Step One PCR system (Applied Biosystems USA) employing Ag Path-ID one-step RT-PCR kit (Thermo Fisher, USA) following the mentioned amplification profile: one cycle of reverse transcription reaction at 50°C for 30 min, initial denaturation of 95°C for 10 min and 40 cycles of two-step PCR at 95°C for 15 s and 60°C for 1 min. The reaction was carried out using 20 nM of forward and reverse primers and 50 nM probe along with 1.5 μl (40 ng) of RNA template in a 25 μl of the total reaction volume.

### Analytical Sensitivity of Real-Time RT-PCR

The analytical sensitivity of the RT-qPCR assay was evaluated using the RNA transcripts mentioned above. The RNA transcript was 10-fold serially diluted ranging from 10^6^ to 10^−2^ copies to obtain the standard curve.

### Specificity of RT-RPA Assay

The specificity of the real-time RT-RPA assay was evaluated employing the RNAs of closely related viruses such as Yellow fever virus, Dengue virus, Japanese Encephalitis virus, Saint Louis Encephalitis virus, Chikungunya virus, Ross River virus, Measles virus, Mumps virus, and Rubella virus.

### Evaluation of Real-Time RT-RPA Assay With Human Clinical Samples

The diagnostic applicability of real-time RT-RPA assay for the detection of WNV infection was evaluated by testing 110 human clinical samples. The results of the real-time RT-RPA assay was compared with CDC reported RT-qPCR (Lanciotti et al., 2000). The clinical sensitivity, specificity, negative predictive values (NPV), and positive predictive values (PPV) of the real**-**time RT-RPA assay were compared with CDC reported RT-qPCR using MedCalc Statistical Software (http://www.medcalc.org). The CDC RT-qPCR positive sample was considered as a true positive. The level of agreement between real-time RT-RPA and RT-qPCR assays was assessed employing the kappa (*k*) coefficient with 95% confidence levels (Cohen, 1960).

## Results

### Optimization of Basic RT-RPA Assay

**Figure 1:**
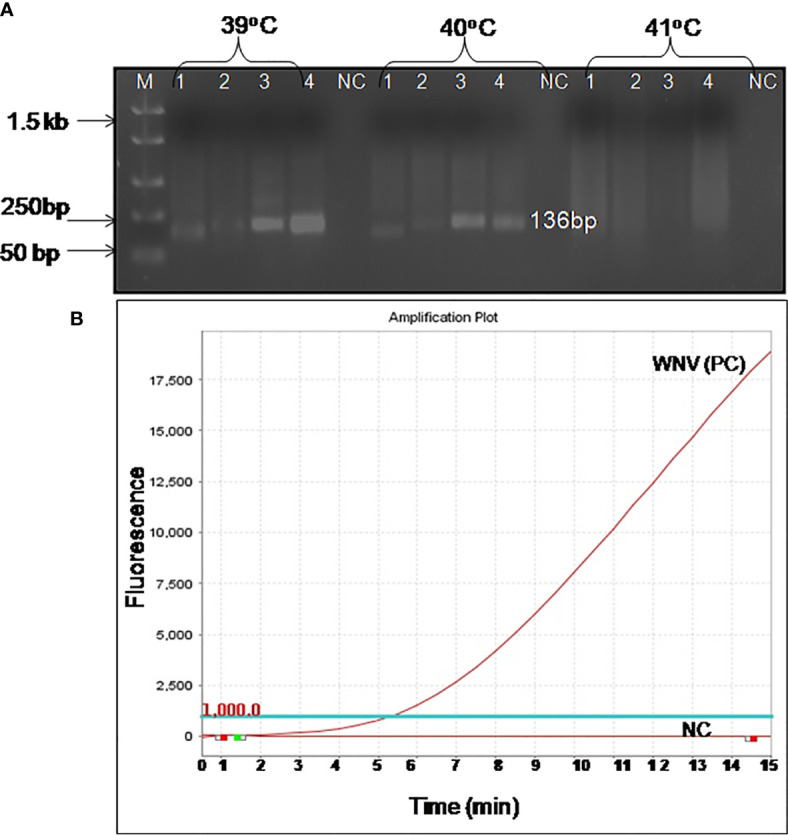
Optimal detection conditions of reverse transcription recombinase polymerase amplification (RT-RPA) assay. **(A)** Basic RT-RPA assay run with different sets of the designed primers and temperatures (39°C, 40°C, and 41°C). M-DNA Marker, Lanes 1–4, RPA amplicons from reactions incubated for 30 min, NC-Negative control. **(B)** Optimization of real-time RT-RPA assay. Optimal primers and probe combinations for the amplification of the WNV env gene by real-time RT-RPA assay.

For the development of WNV-specific real-time RT-RPA assay, four primer sets were designed. To identify the best primer set, it was tested on RNA of WNV at temperatures ranging from 39 to 41°C at 1°C increment for 10, 20 and 30 min. Out of the four **sets**, two sets (set 3 and set 4) were able to amplify WNV RNA efficiently within 30 min at temperatures 39 and 40°C. However, set 4 was able to amplify WNV-RNA efficiently as evident by the high**-**intensity band (136 bp) at 39°C as compared to 41°C (**Figure 1A**). Therefore 39°C temperature and set 4 primers were chosen for the real-time RT-RPA assay to perform further experiments.

### Optimization and Specificity Testing of Real-Time RT-RPA Assay

**Figure 2:**
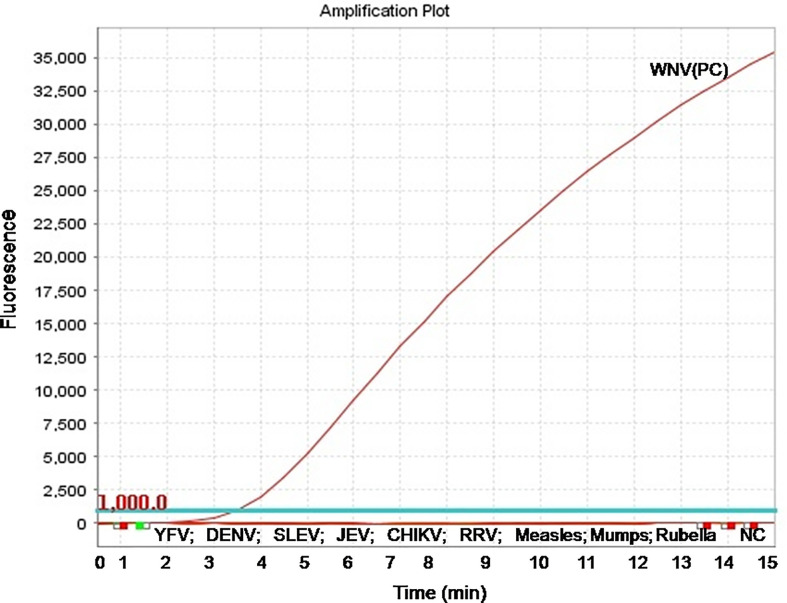
Specificity test results of real-time reverse transcription recombinase polymerase amplification (RT-RPA) assay. The assay was tested for the specificity with RNA of Yellow fever virus, Dengue virus, Saint Louis Encephalitis virus, Japanese Encephalitis virus, Chikungunya virus, Ross River virus, Measles virus, Mumps virus, and Rubella virus.

The real-time RT-RPA assay was also optimized at 30 cycles of 39°C for 15 min in a real-time thermal cycler using the set 4 primers and probe designed within the amplification region of set 4 primers (**Figure 1B**). The specificity of the real-time RT-RPA assay was determined by ruling out the cross**-**reactivity study with closely related viruses. The assay revealed a high degree of specificity for several strains of WNV (**Figure S1**). The closely related viruses namely, Flaviviruses, Alphaviruses and Morbillivirus tested including Yellow fever virus, Dengue virus, Japanese Encephalitis virus, Saint Louis Encephalitis virus, Chikungunya virus, Ross River virus, Measles virus, Mumps virus and Rubella virus showed no amplification with real-time RT-RPA assay (**Figure 2**).

### Comparative Sensitivities of Real-Time RT-RPA and Real-Time RT-PCR Assay

**Figure 3:**
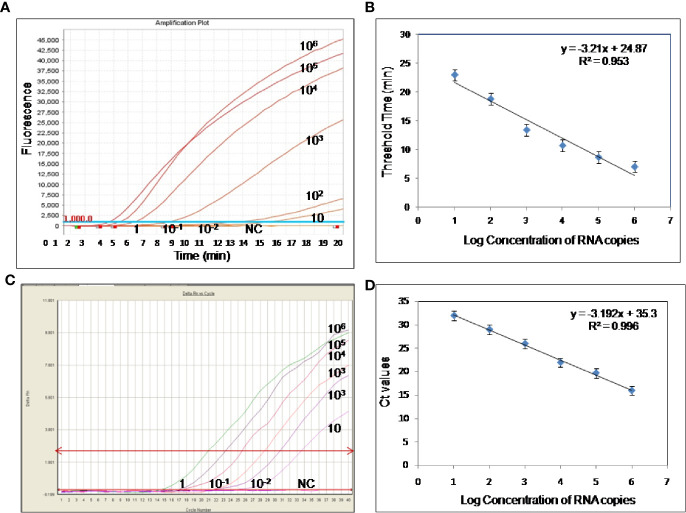
Sensitivity evaluation of real-time reverse transcription recombinase polymerase amplification (RT-RPA) and RT-qPCR assay using tenfold serial dilution of West Nile virus (WNV) targeted *in-vitro* transcribed RNA. **(A)** Amplification plot of real-time RT-RPA assay **(B)** Standard curve for real-time RT-RPA assay plotted between the threshold time values and log concentration of RNA copies **(C)** Real-time kinetics of real-time RT-PCR **(D)** Standard curve for real-time RT-PCR assay plotted between the Ct values and log concentration of RNA copies.

To evaluate the analytical sensitivity of the real**-**time RT-RPA assay with RT-qPCR, tenfold serial dilutions ranging from 10^6^ to 10^−2^ were utilized in triplicates. The analytical sensitivity of both real**-**time RT-RPA and RT-qPCR was found to be 10 RNA copies. The standard curve generated by both real**-**time RT-RPA and RT-qPCR was linear, generating a coefficient of correlation R^2^ = 0.953 (slope, −3.28) and R^2^ = 0.996 (slope, −3.19) respectively (**Figures 3A–D**). The real-time RPA assay had a run time of about 4–14 min for 10^6^–10^1^ copies, respectively, while the RT-qPCR with Ct values between 21 and 34 required about 82-108 min for the final results. The present findings suggest that the turnaround time of real-time RPA assay is about 10–20 times faster than the RT-qPCR (**Table 3**).

**Table 3:** Comparative evaluation of the sensitivity performance and turnaround time between Real-time reverse transcription recombinase polymerase amplification (RT-RPA) and CDC reported Real-time RT-PCR assay.

Copies/reaction	Real-time RT-RPA		CDC reported Real-time RT-PCR	
	Ct	Reaction time, Rt (min)	Ct	Amplification time (min)
10^6^	7.99	3.99	21.2	82.4
10^5^	8.73	4.36	24.3	88.6
10^4^	10.76	5.38	26.4	92.8
10^3^	15.37	7.68	29.3	98.6
10^2^	24.89	12.44	31.5	103.0
10^1^	28.98	14.49	34.2	108.4

### Comparative Evaluation of Real-Time RT-RPA With Real-Time RT-PCR Assay on Clinical Samples

**Figure 4:**
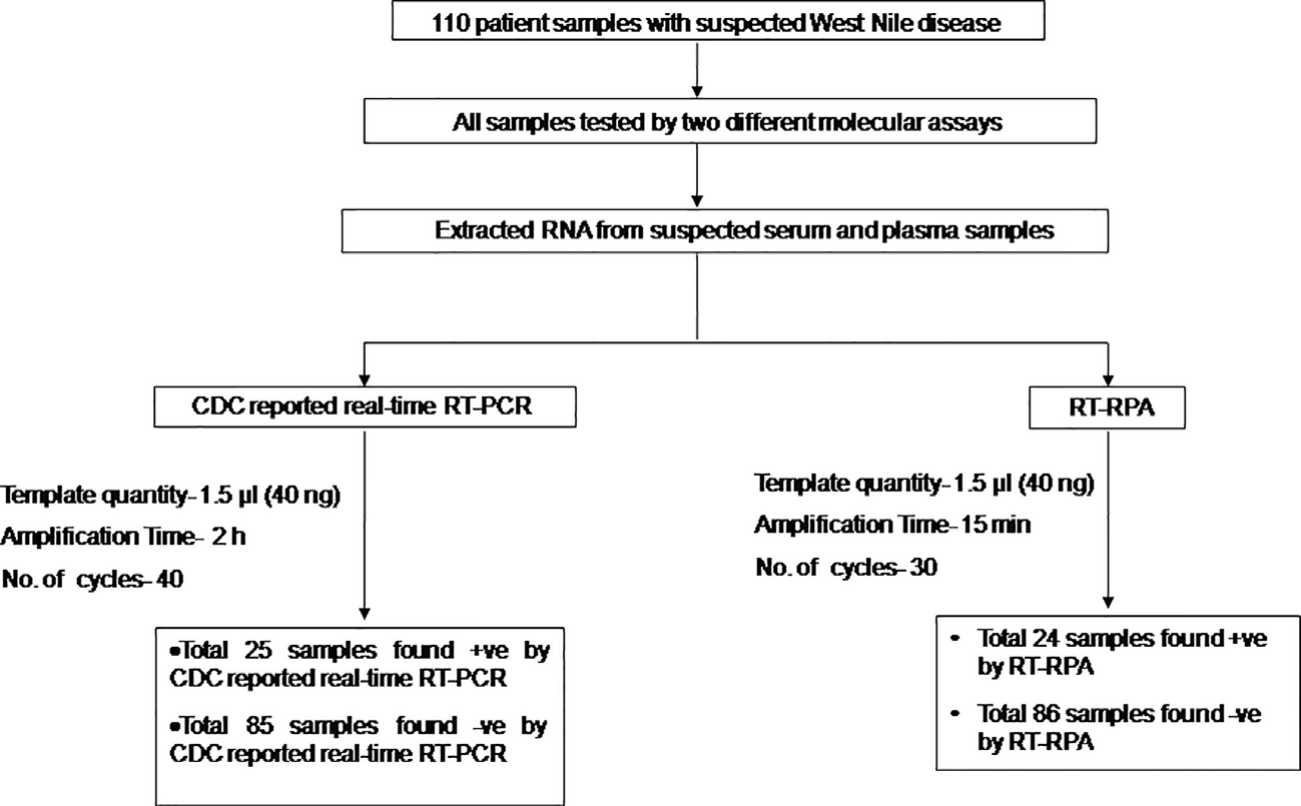
Schematic of the reverse transcription recombinase polymerase amplification (RT-RPA) comparative evaluation study with West Nile suspected human clinical samples.

The diagnostic application of the real-time RT-RPA assay in clinical samples was determined by screening 110 WN suspected serum and plasma samples. Out of 110 samples, 24 samples were found positive and the remaining 86 were found negative by real**-**time RT-RPA assay. The reaction time (Rt) for the real-time RT-RPA and the amplification time for RT-qPCR of 24 positive samples are depicted in **Table 4**. A panel of positive Chikungunya serum samples (n=20) along with healthy subjects (n=30) were also included as the negative control in this study. One sample out of a total of 25 true positives was found negative by the RT-RPA, and the possible reason could be the low level of the target (Ct 35.2) in the sample. Compared to RT-qPCR, the clinical sensitivity of the real**-**time RT-RPA was 96.15% (CI, 80.36% to 99.90%) and the specificity of 100% (CI, 95.07% to 100.00%), with a NPV and PPV of 98.65 and 100% respectively. The level of agreement of RT-RPA assay was found to be 97% for real**-**time RT-RPA (*k*=0.970; strength of agreement: almost perfect) when compared with RT-qPCR (**Table 5**). The findings of the present study are presented in **Figure 4**. The RT-RPA assay required a total of 15 min, compared to 2h for the reference RT-qPCR.

**Table 4:** Detection of West Nile virus (WNV) by reverse transcription recombinase polymerase amplification (RT-RPA) and real-time RT-PCR in clinical samples.

Positive WN samples	Real-time RT-RPA [Reaction time (Rt), min]	Real-time RT-PCR Ct values (amplification time, min)
1	4.3	20.0 (80)
2	5.4	22.0 (88)
3	5.0	28.0 (96)
4	6.6	23.9 (87.8)
5	7.0	26.0 (92)
6	5.0	28.0 (96)
7	5.9	27.8 (95.8)
8	6.0	29.9 (99.8)
9	7.8	27.4 (94.8)
10	5.5	23.6 (87.2)
11	6.2	29.7 (99.4)
12	8.6	28.3 (96.6)
13	8.9	28.5 (97)
14	5.5	25.8 (91.6)
15	9.6	29.7 (99.8)
16	5.3	29.0 (98)
17	6.0	29.8 (99.6)
18	6.4	22.2 (84.4)
19	7.2	21.0 (82)
20	8.1	25.0 (90)
21	7.3	25.9 (91.8)
22	8.8	29.8 (99.6)
23	7.1	21.0 (82)
24	4.0	19.5 (79)

**Table 5:** Diagnostic performance comparison between real-time reverse transcription isothermal recombinase polymerase amplification (RT-RPA) assay for human clinical samples (n =110) and reference CDC reported real-time RT-PCR method.

	Real-time RT-RPA	CDC reported real-time RT-PCR
Positive	24	25
Negative	86	85
Sensitivity	96.15% 100% 100% 98.65% 97.0% (*k* =0.970, almost perfect agreement)	
Specificity		
PPV		
NPV		
Agreement (*k* value)		

## Discussion

Outbreaks of WNV in human beings have been reported in Africa, West and South Asia, North America, the Middle East, Europe, and Australia (Lanciotti et al., 1999). WNV emerged into North America in 1999 (late summer) when many cases of WN disease were reported in New York City. In 2000, the West Nile infection was detected in many states of the USA (Marfin and Gubler, 2001). Millions of cases were reported between 1999-2010, with over 12,000 identified cases of meningitis or encephalitis and more than 1,300 deaths (Kilpatrick, 2011).

A number of various serological diagnostic methods are used for the detection of WNV in clinical specimens comprising ELISA, PRNT, virus isolation and Immunofluorescence test, etc. All of these methods are tedious and time-consuming (Wong et al., 2004). Recently, many researchers have developed molecular-based systems for the detection of WNV in clinical samples that were negative for virus isolation, suggesting the utility of molecular-based assays for the detection of WNV infection. Several PCR-based assays including conventional RT-PCR, SYBR Green RT-PCR and TaqMan real-time RT-PCR have been developed for the rapid diagnosis of WNV (Vanlandingham et al., 2004; Kumar et al., 2014). The real-time PCR-based assays are superior to conventional RT-PCR methods in terms of rapidity, sensitivity, quantitative measurement, specificity with lower contamination rate. The development of probe-based real-time has eliminated the post-PCR processing i.e. agarose gel electrophoresis (Lanciotti et al., 2000). Despite the high degree of amplification, these PCR-based assays are costly, time-consuming, labor-intensive, and need technical expertise to perform and complex to adapt for use in clinics. It is, therefore, crucial to overcome these limitations and require a sensitive, specific, rapid and cost-effective technique for the screening of new WNV strains (Lanciotti and Kerst, 2001). Two LAMP assays have been developed for the detection of WNV. The LAMP assay utilized four to six primers and results can be usually analyzed visually after 60 min (Parida et al., 2004; Cao et al., 2016). Recent LAMP assays development begins to reveal shorter incubation time (Kurosaki et al., 2016). But LAMP assay has its own limitations of requiring six sets of primers. In contrast, the isothermal gene amplification method can be a better alternative to PCR-based detection assays in clinical settings.

The newly reported isothermal assay RPA obviates the need for costly instrumentation and reaction can be incubated in a dry bath or water bath. The assay requires two primers and one probe and three enzymes. Apart from this, RPA reagents are supplied in a lyophilized form stable at room temperature (25°C–38°C) (Faye et al., 2015; Daher et al., 2016); which permits independence from the cooling chain. But, designing of RPA probe and primers are still difficult, as several sets of primers must be checked for the selection of a working set, as there is no suitable available software so far. RPA has several important advantages over PCR methods, the first being that it does not require a pure template for the analysis, which is required for PCR-based assays (Mekuria et al., 2014; Li et al., 2019). The second advantage is that results are available in a much shorter time i.e., 15–20 min for RPA and 1.5-3.0 h for PCR assays. The third advantage is that it is relatively easy to perform as it comes with the ready-to-use freeze-dried reagent which makes the operator’s job much easier and also minimizes the pipetting errors (Daher et al., 2016). The RPA assay has also some important advantages over other isothermal techniques such as LAMP, as it uses shorter primers and makes use of probes which enhance the specificity of the assay (Daher et al., 2016). When comparing the practical applicability of RPA, it is the easiest to perform when compared with PCR and LAMP techniques and also its reagents are very stable.

The present research work was intended to develop a real**-**time RT-RPA assay for the rapid detection of WNV targeting env gene. The RT-RPA assay developed here is rapid, as the result is obtained within 15 to 20 min. The assay could detect as low as 10 RNA copies supporting the utility for the detection of infection in samples with low viremia. The real-time RT-RPA assay was tested further for specificity using the RNAs of closely related viruses such as Yellow fever virus, Dengue virus, Japanese Encephalitis virus, Saint Louis Encephalitis virus, Chikungunya virus, Ross River virus, Measles virus, Mumps virus, and Rubella virus and no cross-reaction was observed, suggesting that the assay is highly specific.

The utility of the real-time RT-RPA assay for the WNV disease diagnosis was determined by testing 110 samples from patients with retinitis, uveitis, febrile illness (between day 1 to day 9). RPA was reported to detect extensive human pathogens including viruses, bacteria, parasites and fungi. RPA has so far been used for the detection of several infectious agents including Chikungunya virus, Dengue, Yellow fever, Ebola, Rift Valley Fever and CCHF viruses, etc (Faye et al., 2015; Kurosaki et al., 2016; Wahed et al., 2015; Patel et al., 2016). To the best of our understanding, we present for the first time the development of a real-time RT-RPA assay for simple, rapid, sensitive, and specific detection of WNV in human clinical samples. Compared to RT-qPCR, the clinical sensitivity of the real**-**time RT-RPA was 96.15% (CI, 80.36% to 99.90%) and the specificity of 100% (CI, 95.07% to 100.00%), with a NPV and PPV of 98.65% and 100% respectively. The level of agreement of RT-RPA assay was found to be 97% for real**-**time RT-RPA (*k*=0.970; strength of agreement: almost perfect) when compared with RT-qPCR. The strength of the RPA assay lies in its rapidity, the present real-time RPA had provided results with similar sensitivity and specificity in much lesser time. The run time of the real-time RT-RPA assay was about 4 to 14 min for 10^6^ to 10^1^copies, respectively, while the RT-qPCR with Ct values between 21 and 34 required about 82 to 108 min to obtain the final results. Similarly, when it comes to the screening of clinical samples, the real-time RT-RPA has provided almost similar findings to that of RT-qPCR within a short time period (turnaround time of real-time RPA assay is about 10 to 20 times faster than the RT-qPCR) which confirms its utility in the rapid and sensitive diagnosis of WNV infection. The present study suggests that the real-time RT-RPA assay is an important emerging tool for the real-time and rapid detection of WNV. There are several studies where RPA assay was developed for the detection of various viruses and bacteria using the real-time PCR instrument (Yang et al., 2016; Kim and Lee, 2017; Yang et al., 2017a; Yang et al., 2017b). In the present study also we used a real-time PCR machine available in our laboratory. RPA assay is highly flexible as it can be adapted to various detection systems. A simple fluorescence tube scanners (ESEQuant, Qiagen; Genie, OptiGene; T8-ISO, TwistDX) which are simpler, cost-effective, and are battery operated can be used in the field as compared to the expensive and lab-based real-time thermal cyclers (James and Macdonald, 2015; Wahed et al., 2015; Daher et al., 2016).

In conclusion, the real**-**time RT-RPA assay described here is a simple, specific, and sensitive method for the detection of WNV. The assay has the potential to accurately screen a large number of clinical samples within 15 min of run time. The present real**-**time RT-RPA assay can prove to be a feasible alternative to laboratory-based RT-PCR assays for detection of the WNV in endemic regions and poor health care settings.

## Data Availability Statement

The original contributions presented in the study are included in the article/**Supplementary Material**. 

## Ethics Statement

The present study was approved by the Institutional Ethical Committee (IBSC/12/VIR/JSK/18).

## Author Contributions

JK and SK conceived and designed the experiments. PT, SK, and JK performed the experiments. JK, SK, and SP analyzed the data. JK, SK, and PT wrote the paper. All authors contributed to the article and approved the submitted version.

## Funding

This work was supported by Defence Research and Development Organization (DRDO) research funds and PT is a recipient of DRDO-SRF fellowship.

## Conflict of Interest

The authors declare that the research was conducted in the absence of any commercial or financial relationships that could be construed as a potential conflict of interest.

## Supplementary Material

Supplementary Figure 1 Details of the RPA amplicon for WNV. The nucleotide alignment matching in different strains of WNV. Primers and probe sequences are bold, highlighted and underlined. WNV RPA forward primer and probe is presented in sense, and the reverse primer is presented as reverse complement sequences.

Supplementary Table 1 Viral strains used for the evaluation of specificity of the RT-RPA assay.
