# Supplementary material for: Development and Evaluation of Real-Time Reverse Transcription Recombinase Polymerase Amplification Assay for Rapid and Sensitive Detection of West Nile Virus in Human Clinical Samples
Source: Front Cell Infect Microbiol. 2021 Feb 23;10:619071. doi: 10.3389/fcimb.2020.619071 (PMC7940365; doi:10.3389/fcimb.2020.619071)
Supplement: Supplementary Table 1 — Viral strains used for the evaluation of specificity of the RT-RPA assay. [file Table_1.docx]

**Table S1. Viral strains used for the evaluation of specificity of the RT-RPA assay**

| West Nile virus Eg101 strain, Yellow Fever virus 17D strain, Dengue virus ThNH7/93 strain, Japanese Encephalitis virus JaOArS982 strain, Saint Louis Encephalitis virus parton strain, Chikungunya virus DRDE07 strain, Ross River virus T48 strain, Measles virus Edmonston-Zagreb strain, Mumps virus L-Zagreb strain, and Rubella virus Wistarra 27/3 strain |
| --- |
